# Angiogenic Endothelial Cell Signaling in Cardiac Hypertrophy and Heart Failure

**DOI:** 10.3389/fcvm.2019.00020

**Published:** 2019-03-06

**Authors:** Rajinikanth Gogiraju, Magdalena L. Bochenek, Katrin Schäfer

**Affiliations:** ^1^Center for Cardiology, Cardiology I, Translational Vascular Biology, University Medical Center Mainz, Mainz, Germany; ^2^Center for Thrombosis and Hemostasis, University Medical Center Mainz, Mainz, Germany; ^3^Center for Translational Vascular Biology, University Medical Center Mainz, Mainz, Germany; ^4^Deutsches Zentrum für Herz-Kreislauf-Forschung e.V., Partner Site RheinMain (Mainz), Mainz, Germany

**Keywords:** angiogenesis, endothelial cells, fibrosis, heart failure, hypertrophy, p53, PTP1B

## Abstract

Endothelial cells are, by number, one of the most abundant cell types in the heart and active players in cardiac physiology and pathology. Coronary angiogenesis plays a vital role in maintaining cardiac vascularization and perfusion during physiological and pathological hypertrophy. On the other hand, a reduction in cardiac capillary density with subsequent tissue hypoxia, cell death and interstitial fibrosis contributes to the development of contractile dysfunction and heart failure, as suggested by clinical as well as experimental evidence. Although the molecular causes underlying the inadequate (with respect to the increased oxygen and energy demands of the hypertrophied cardiomyocyte) cardiac vascularization developing during pathological hypertrophy are incompletely understood. Research efforts over the past years have discovered interesting mediators and potential candidates involved in this process. In this review article, we will focus on the vascular changes occurring during cardiac hypertrophy and the transition toward heart failure both in human disease and preclinical models. We will summarize recent findings in transgenic mice and experimental models of cardiac hypertrophy on factors expressed and released from cardiomyocytes, pericytes and inflammatory cells involved in the paracrine (dys)regulation of cardiac angiogenesis. Moreover, we will discuss major signaling events of critical angiogenic ligands in endothelial cells and their possible disturbance by hypoxia or oxidative stress. In this regard, we will particularly highlight findings on negative regulators of angiogenesis, including protein tyrosine phosphatase-1B and tumor suppressor p53, and how they link signaling involved in cell growth and metabolic control to cardiac angiogenesis. Besides endothelial cell death, phenotypic conversion and acquisition of myofibroblast-like characteristics may also contribute to the development of cardiac fibrosis, the structural correlate of cardiac dysfunction. Factors secreted by (dysfunctional) endothelial cells and their effects on cardiomyocytes including hypertrophy, contractility and fibrosis, close the vicious circle of reciprocal cell-cell interactions within the heart during pathological hypertrophy remodeling.

Heart failure is a major cause of morbidity and mortality affecting several million individuals worldwide. Left ventricular hypertrophy represents an adaptive response of the heart to an increased workload but is also a well-established risk factor for cardiovascular mortality (1) and may progress to ventricular dilation and heart failure, if untreated. Among the factors contributing to disease progression and cardiac decompensation, findings in patients, and preclinical models underline the critical role of defects in cardiac angiogenesis and vascularization in the transition from adaptive to maladaptive cardiac remodeling. Alterations in hemodynamic and mechanical factors as well as hypoxia stimulate the release of angiogenic growth factors from cardiomyocytes to induce the parallel growth of the supplying vasculature. *Vice versa*, activated or dysfuntional endothelial cells may also affect the function of other cell types in the heart, including myocytes and non-myocytes. However, these mechanisms fail to stimulate capillary growth in advanced stages of the disease. In this review article, we will explore possible causes underlying defects in angiogenic signaling in the hypertrophied heart resulting in a mismatch between cardiac oxygen needs and supply. Although myocardial ischemia also is associated with hypertrophy of the surviving myocardium, we will focus primarily on cardiac hypertrophy developing in response to increased pressure, but also volume overload.

## Angiogenesis in the Heart: a Brief Overview

In humans and other mammalian species, the heart is perfused via the coronary circulation, in contrast to amphibians, in which the spongy heart musculature is lined by a thin monolayer of endothelial cells and directly perfused with blood from the ventricular cavity (2). During ontogenic development, changes in heart size are accompanied by the gradual transformation into a compact myocardium supplied through coronary vessels. In humans, myocardial perfusion is achieved by large epicardial coronary conductive arteries (diameter >500 μm), which further divide and branch into prearterioles (diameter 500–100 μm), intramyocardial arterioles (diameter <100μm) and finally, capillaries (3). Due to their extramyocardial position and wall thickness, epicardiac vessels are not under direct vasomotor control by metabolites released from the myocardium. Instead, cardiac oxygen consumption and supply is balanced on the level of the intramyocardial arterioles and capillaries.

Numerous bifurcations and anastomoses between capillaries create a dense vascular network intimately entwining the cardiomyocyte fibers in a range allowing the diffusion of nutrients and oxygen (4). Early histological quantitative analyses of rat, cat, and human hearts revealed at least one capillary for each muscle fiber in all parts of the hearts, except the auricular walls and the Purkinje fibers, where the capillary density was found to be less abundant (5). Moreover, capillary density did not differ between the left and right ventricle or the interventricular septum (5). In fact, no cardiac myocyte is more than 2–3 μm away from a cardiac microvascular endothelial cell (6). Capillary density is higher in infants and decreases during postnatal heart growth, but thereafter remains constant (7). Of note, aging *per se* is associated with cardiac microvascular rarefaction as well as other important changes at the level of the terminal vascular bed, as shown in mice (8).

Regarding other parameter affecting cardiac perfusion: Earlier comparisons of different species, including “athletic” (e.g., hare or wild rat) and sedentary (e.g., rabbit or laboratory rat) animals, revealed that cardiac capillary density is inversely related to heart rate with high-frequency having a less dense capillary network (9). Brachycardia improves cardiac perfusion by favoring diastolic filling and coronary perfusion and also by reducing cardiac oxygen demands. From a therapeutic standpoint, prolongation of the diastolic interval achieved by bradycardial pacing in rabbits (10) and pigs (11) or by administration of the K_ATP_ channel antagonist and selective sinus blocking drug alinidine to rats (12) was shown to induce angiogenesis in normal hearts and to increase the capillary density without affecting cardiomyocyte size or heart weight. Similar proangiogenic effects of long-term brachycardia were observed in hearts with comprised vascular supply due to ischemic or hypertensive damage (13). The angiogenesis-promoting effects of brachycardia may be triggered by increased mechanical stretch and vessel wall tension as a result of the increased stroke volume capacity of the heart (14), an important mechanism of angiogenic growth factor release (15, 16). In line, the proangiogenic effects of cardiac β-adrenoreceptor blockade in rats could be reduced by administration of a decoy vascular endothelial growth factor (VEGF) receptor (Ad-Flk) (17). The positive lusitrophic effects of endothelial cell-derived nitric oxide (NO) resulting in the earlier onset of relaxation and a longer diastole (18) might also play a role in the stimulation of cardiac angiogenesis, or its absence in case of endothelial dysfunction (19).

## Vascular Changes During Cardiac Hypertrophy and Heart Failure

Rapid heart growth is observed during early postnatal development, whereas later in life, myocardial hypertrophy develops as adaptive response of the heart to chronically increased workload in order to maintain cardiac output. Any increase in heart tissue must be matched by a corresponding expansion of the coronary vasculature to maintain an adequate supply of oxygen and nutrients. Short-term regulatory mechanisms activated by inadequate oxygenation include adenosine-induced vasodilation to maintain perfusion. If the stimulus persists, hypertrophied cardiomyocytes and other cell types in the heart secrete factors to stimulate the parallel growth of their supplying vascular network in order to meet the increased oxygen demands. Important angiogenic mediators in the heart will be discussed in one of the next sections.

In cardiac hypertrophy developing in response to postnatal growth, physical exercise or pregnancy, so-called physiological hypertrophy, capillaries grow proportional to cardiomyocyte volume thus maintaining the capillary density observed in normal non-hypertrophied hearts. In contrast, maladaptive or pathological cardiac hypertrophy is characterized by an inadequate rarefaction of the cardiac microvasculature. Since cardiac perfusion and blood supply is critically determined on the level of the capillaries (20), any reduction in capillary density will result in cardiac underperfusion. The insufficient oxygen and nutrient supply despite the increased metabolic needs of the hypertrophic cardiac muscle may cause hypoxia, cardiomyocyte death and fibrosis, characteristic findings in pathological hypertrophy (21). In fact, the imbalance between capillary and myocardial fiber growth is considered to be an important contributor to the transition from hypertrophy to heart failure (22).

Changes in the cardiac microvasculature during cardiac hypertrophy have been examined in a number of studies, for example in hypertrophic (23) and dilated cardiomyopathy (24) or hypertensive heart disease (25). The reduced capillary density observed in patients with hypertrophic cardiomyopathy may also be responsible for the clinical signs and symptoms of ischemia in the absence of epicardial coronary artery stenosis (26). The independence of myocardial capillary density from the presence of coronary artery disease was also documented in transmural left ventricular autopsy specimens of 124 patients with heart failure with and without coronary artery disease (27). In addition to structural alterations of the cardiac microvasculature, other factors determining cardiac perfusion, such as the length of the diastole, myocardial distensibility, or the capacity of the myocardium to overcome arteriolar resistance, are also frequently impaired in patients with heart failure (28).

The reduction of myocardial blood flow due to left ventricular hypertrophy results in perfusion deficits and myocardial ischemia at times of elevated cardiac stress, in particular in the subendocardium, as shown in dogs (29, 30). Already under physiological conditions, the capillary density in the heart exhibits a transmural decline being higher in the subepicardium than in the subendocardium (31) predisposing this area to ischemia during stress. In line, patients with hypertrophic cardiomyopathy and normal coronary arteries show symptoms, electrocardiographical signs and other pathological findings consistent with subendomyocardial ischemia (32).

Interestingly, capillary density with left ventricular hypertrophy has been shown to depend on the age of onset. For example, vascular rarefaction was present in adults with acquired aortic stenosis, whereas capillaries were found to grow proportional to cardiomyocyte volume in patients with congenital aortic stenosis and aortic coarctation (7).

Comorbidities or cardiovascular risk factor exposure may promote cardiac hypertrophy and reduce cardiac contractility via impaired production of NO from dysfunctional endothelial cells (19). In healthy endothelium, stretch or shear stress stimulates the release of NO (33), which may act on adjacent smooth muscle cells to induce vasodilation or on cardiomyocytes to increase contractility. For example, cardiac NO was shown to increase the left ventricular diastolic compliance (34), and this effect was shown to be mediated by cyclic GMP-dependent protein kinase G activation, troponin phosphorylation resulting in reduced myofilament Ca^2+^ sensitivity (35). Treatment of DAHL salt-sensitive rats with hypertension-induced heart failure with the endothelial NO synthase (eNOS) enhancer AVE3085 attenuated diastolic dysfunction, cardiac hypertrophy and fibrosis (36), and similar findings were observed using the NO donor LA419 (37). Also, the beneficial effects of endothelial progenitor cells on cardiac hypertrophy, fibrosis, and angiogenesis were found to depend on the expression of eNOS in the bone marrow (38). Conversely, inhibition of NO production in cardiomyocytes resulted in a rapid increase in the formation of reactive oxygen species, p38 MAP kinase activation and enhanced TGFβ and TNFα expression, and similar findings were observed in eNOS-deficient mice (39).

## Findings in Preclinical Models of Cardiac Hypertroph*y*

Similar to observations in patients, morphometric studies in preclinical models of cardiac hypertrophy also revealed a reduction in capillary density and inadequate growth of the coronary microvasculature [reviewed in Kamo et al. (40)]. Cardiac angiogenesis as adaptive response to chronic pressure overload is not restricted to the left heart, but also observed in the right ventricle in response to pulmonary arterial hypertension [reviewed in Ryan and Archer (41)]. Similar to left ventricular hypertrophy, increased angiogenesis is only observed in the early stages, whereas continued exposure to hypoxia was shown to lead to progressive right ventricular hypertrophy without additional angiogenesis (42).

A causal role of angiogenesis defects for the conversion from compensated to decompensated hypertrophy is suggested by experimental findings that inhibition of blood vessel formation using angiogenesis inhibitors like TNP-470 or a VEGF trap accelerated the development of cardiac dysfunction (43–45), whereas angiogenesis stimulation delayed the onset of heart failure (46). Interestingly, angiogenesis itself may provoke a hypertrophic response of the myocardium in the absence of external prohypertrophic stimuli. For example, mice with cardiomyocyte-specific overexpression of the angiogenic factor PR39 not only exhibited a dense cardiac capillary network, but also developed cardiac hypertrophy, and heart growth could be diminished by treatment with the eNOS inhibitor L-NAME (47). Similarly, inducible expression of the proangiogenic transcription factor hexamethylene-bis-acetamide-inducible protein-1 in cardiomyocytes of adult mice was associated with increased vascularization, myocardial growth and expression of transcription factors involved in coordinated, physiological hypertrophy and improved cardiac output (48). In this regard, the simultaneous application of factors involved not only in the regulation of cardiac angiogenesis, but also in cardiomyocyte growth and contractility has been successfully tested in preclinical models and was found to provide a greater therapeutic benefit in preventing or postponing heart failure than a single molecule approach (49). On the other hand, more cardiac vessels do not necessarily translate into cardiac protection: Conditional endothelial overexpression of peroxisome proliferator-activated receptor-β/δ enhanced cardiac angiogenesis and cardiomyocyte growth, but did not protect against chronic ischemic injury following myocardial infarction (50).

Direct comparisons revealed that cardiac hypertrophy experimentally induced by transverse aortic constriction (TAC) resulting in chronically increased pressure overload was associated with a reduced capillary density, whereas capillary angiogenesis was maintained in hearts exposed to volume overload induced by aortocaval shunt (51, 52) or cardiomyocyte hyperplasia induced by genetic overexpression of cyclin D2 (53). Moreover, cardiac hypertrophy in response to transaortic banding was associated with cardiac fibrosis and apoptosis, whereas aortic regurgitation was associated with angiogenesis (54). Although mean wall stress was similar in both models, systolic wall stress was significantly increased in TAC and diastolic wall stress was mainly elevated in aortic regurgitation. Of note, the model of transverse (supravalvular) aortic constriction commonly used to experimentally induce increased cardiac pressure overload in experimental animals pathophysiologically differs from valvular aortic stenosis in patients, because coronary artery perfusion is intact in TAC, but impairced in aortic stenosis.

## Quantitative Assessment of Coronary Angiogenesis in Preclinical Models

Microvessel density in the heart is often quantified by counting the number of capillaries per cardiomyocyte fiber (C/F ratio) on transverse tissue sections. However, this methodology detects alterations in tissue oxygenation only if cardiomyocyte size does not change. In the case of cardiomyocyte hypertrophy, no changes in the C/F ratio would still result in an increased oxygen diffusion distance and possibly hypoxia. Measurement of the intercapillary distance may provide additional information. An additional means to assess cardiac vascularization is the quantification of the capillary density (i.e., numbers of capillaries per square mm), although this method may be subject to technical variables (e.g., tissue shrinkage during fixation). Image acquisition and analysis software may be used in addition to the manual quantification of vessel numbers to determine additional parameters, such as the average vessel length or diameter or the number and complexity of branch points, to better describe spatial vascular patterns in the heart. Platelet-endothelial cell adhesion molecule (PECAM or CD31) is a commonly used marker to stain endothelial cells and has the advantage that immature endothelial cells. i.e., the ones covering newly formed blood vessels, are also detected, while these might be missed if the tight junction protein VE-cadherin or the Weibel-Palade body component von Willebrand factor (vWF) are chosen as endothelial cell markers. However, it should be kept in mind that CD31 is also expressed on hematopoietic cells (55). Staining for enzymes expressed in endothelium, such as alkaline phosphatase, may be used to visualize viable endothelium, but is also not specific for endothelial cells. To simultaneously stain endothelial cells and determine microvessel perfusion, intravital perfusion (i.e., via coronary arteries in large and via the left atrium in smaller animals) with an endothelial-binding lectin is a valuable tool and may be adapted to other dyes to assess the extent of the capillary bed in the beating heart (5). By using fluorescent dyes coupled to proteins of different sizes a simultaneous analysis of the intravasal and extracellular space is also possible (56). Of note, non-perfused capillary endothelial cells may be missed with this method, especially if not enough time is allowed for complete perfusion, and the capillary density may be underestimated. Visualization of hypoxia (e.g., using hypoxyprobe™ or the HIF1α target gene carboxyanhydrase IX) as functional estimates or readout of myocardial tissue oxygenation may also be considered. State-of-the-art microscopy techniques including light-sheet, scanning electron or confocal microscopy may help to improve the resolution and differentiation of endothelial from other cell types and their structural integration into the heart. Potential targets for molecular imaging of cardiac angiogenesis [reviewed in Mandic et al. (57)] may be employed to obtain more detailed insights into specific functional alterations during cardiac remodeling processes.

## Growth Factor Signaling Involved in the Regulation Cardiac Angiogenesis

As outlined above, capillary density is an important factor in the development of either physiological or pathological hypertrophy. But what are the factors regulating angiogenesis in the heart? In general, capillary perfusion, the length of the diastolic interval, and diastolic ventricular chamber filling provide initiating stimuli for capillary angiogenesis during cardiac enlargement (58). In addition, the mechanisms and cell types involved in cardiac angiogenesis may depend on the time of onset (embryonic development, early postnatal heart growth, age) as well as the stimulus (training, ischemia, mechanical forces).

### Vascular Endothelial Growth Factor

During cardiac hypertrophy, angiogenic growth factors are released from stretched or hypoxic cardiomyocytes and other cells, which interact with their specific receptors on neighboring endothelial cells. The angiogenic growth factor VEGF is critically involved in cardiac vascularization and function, as shown in transgenic mice lacking all VEGF-A isoforms in cardiomyocytes resulting in coronary hypovascularization, ventricular thinning and contractile dysfunction (43). Despite the reduced cardiac vascularity, cardiomyocyte necrosis or fibrosis were not observed, although mice were not subjected to any type of cardiac injury in this study. Mice lacking only the VEGF_164_ and VEGF_188_ isoform exhibited impaired myocardial angiogenesis resulting in ischemic cardiomyopathy and heart failure (59). Capillaries in hearts of these mice also were found to be more irregular, tortuous and dilated, indicative of incomplete vessel remodeling. In this regard, VEGF initiates blood vessel formation by enhancing vascular permeability and increasing endothelial cell proliferation whereas angiopoietins are subsequently required for further remodeling, maturation and stabilization of the initially immature vasculature (60).

The essential role of VEGF for adjusting the coronary microvasculature to changes in oxygen demands has been demonstrated in mice with inducible expression of a VEGF sequestering soluble receptor (61) or after VEGF blockade via adenoviral overexpression of decoy VEGF receptors (44). Conversely, exogenous administration of VEGF to rabbits after aortic banding was shown to maintain cardiac angiogenesis and to delay decompensation (62). Similarly, intramyocardial gene therapy with VEGF_165_ in rats prevented the rarefaction of the cardiac microvasculature occurring with age and stabilized cardiac vessel density to levels observed in young animals (63). Transgenic mice with a chronic increase in VEGF-A in their hearts exhibited an increased cardiac angiogenesis and developed cardiac hypertrophy resulting in enhanced basal cardiac function with age progression (64). In 179 patients with hypertension and 169 healthy controls polymorphisms in the VEGF-A gene were found to be associated with left ventricular mass and systolic function (65). On the other hand, inhibitors of VEGF signaling clinically approved for the treatment of cancer or angioproliferative eye disease are associated with cardiovascular toxicity (66).

VEGF expression is regulated by mechanical stress and its secretion from cardiomyocytes can be enhanced by stretch (67). Factors involved in the upregulation of VEGF expression in hypertrophied cardiomyocytes include HIF1α (68), but also NFκB (16), TGFβ (69), or endothelin-1 (70), that is factors involved in inflammation and fibrosis signaling. Cardiomyocyte-specific deletion of GATA4, a transcription factor expressed in cardiomyocytes binding to the VEGF-A promoter also exhibited a reduced capillary density in their hearts, while conditional overexpression of GATA4 significantly enhanced cardiac vascularization (71). Of note, the pressure overload-induced cardiac dysfunction in GATA4-null mice could be partially rescued by gene delivery of VEGF and angiopoietin-1. GATA4 gene transfer was also shown to improve cardiac function following myocardial infarction by promoting cardiac angiogenesis and stem cell recruitment and inhibiting apoptosis (72). In fact, upregulation of GATA4 in response to mechanical stimuli has a unique role in the control of cardiac growth by enhancing the transcription of both angiogenic and hypertrophic factors. The regulation of hypertrophic gene expression in the adult heart also involves GATA6 (73, 74), however its regulatory role does not seem to extend to cardiac angiogenesis (75). On the other hand, GATA2 controls angiogenesis in response to mechanical cues (76), among others via the regulation of small non-coding RNAs (miRNAs) such as miR-126 and miR-221, but is not known to be differentially expressed in the hypertrophied heart (77).

VEGF itself may enhance the transcription of other angiogenic growth factors by acetylation of ETS1, a master regulator of endothelial gene transcription (78), and stimulating the pause release of RNA polymerase II from their promoter region (79).

In addition to VEGF-A, a time course analysis in mice following aortic banding revealed that VEGF-C and VEGF-D are upregulated during compensatory hypertrophy, whereas VEGF-B decreased during decompensation and heart failure (80). Adenoviral administration of VEGF-B_186_ resulted in improved angiogenesis, inhibition of apoptosis and cardiomyocyte proliferation and could prevent cardiac decompensation following TAC (80). These findings suggest that reductions in cardiac VEGF-B levels could underlie the vascular rarefaction during advanced stages of cardiac hypertrophy. VEGF-B is also important for cardiac function during diastolic heart failure, as shown in rats infused with angiotensin II for 2 weeks via osmotic minipumps (81). Interestingly, adenoviral VEGF-B transfer did not significantly alter capillary number or density within the heart in this study, and another study reported that VEGF-B resulted in an increased growth of epicardial coronary vessels and intramyocardial arterioles (82). Similar findings were observed in rats overexpressing VEGF-B following myocardial infarction which showed diminished cardiac fibrosis and improved contractility in the absence of a significant induction of angiogenesis (83).

VEGF in the heart may not only be a major regulator of cardiac angiogenesis, but also exert cytoprotective, antioxidative, and antiapoptotic effects on cardiomyocytes (84, 85). Moreover, VEGF may contribute to cardiomyocyte growth, as suggested by findings that VEGF-neutralizing peptides slightly but significantly inhibited the endothelin-1-induced increase in cardiomyocyte cell surface area and ^[14C]^leucine incorporation (70). By acting on both endothelial cells and cardiomyocytes, VEGF upregulation may thus coordinate the growth of both cell types during cardiac hypertrophy, whereas inhibition of VEGF signaling promotes the transition to heart failure [22, 44].

VEGF binds to VEGFR receptor 1 (VEGFR1) and receptor 2 (VEGFR2) expressed in the heart. Binding of VEGF to VEGFR1 leads to activation of cyclic guanosine monophosphate-dependent protein kinase-1 signaling pathways resulting in the formation of new blood vessels and regression of hypertrophy, whereas activation of VEGFR2 signaling results in cardiac hypertrophy (86). Interestingly, both pathways could be independently targeted in cardiomyocytes using miR-374 which inhibited the VEGFR1-mediated regression pathway to promote cardiac hypertrophy (87).

Other miRNAs enriched in endothelial cells indirectly targeting VEGF and other angiogenic growth factors include miR-214 (88, 89) and miR-126 (90). For example, miR-214 expression was found to be upregulated during maladaptive cardiac remodeling in mice and in patients with heart failure (91). Reduced expression of miR-126 in endothelial progenitor cells independently predicted the outcome of patients with chronic heart failure (92, 93). A role for miRNA based gene regulation in the negative control of cardiac angiogenesis during hypertrophy was also demonstrated for miR-124 (main target: the tetraspanin CD151) (94), miR-195a-3p (main targets: CD31, eNOS, VEGF) (95), miR-320 (main targets: IGF-1, Hsp20 and ETS2) (96) and miR-665 (main target: CD34 expressed on hematopoietic and endothelial cells) (97). Importantly, silencing of miR-652 (targeting the Notch1 ligand Jagged1) was found to stabilize cardiac angiogenesis and prevent heart failure in mice with established hypertrophy following aortic banding (98). Regarding the role of long non-coding RNAs (lncRNAs; >200 nucleotides in length) expressed in endothelial cells and involved in the regulation of an angiogenic gene program, such as STEEL (99), LEENE (100), MALAT1 (101), MANTIS (102), MIAT (103), GATA6-AS (104), and others, collectively termed “Angio-LncRs,” their role in the regulation of cardiac angiogenesis during non-ischemic hypertrophy and heart failure has yet to be determined.

Binding of VEGF and other growth factors signaling via tyrosine kinase receptors induces phosphorylation of protein kinase B (Akt), a major signaling pathway in the heart involved in the regulation of cardiac growth, contractile function and angiogenesis (105). In this regard, short-time activation of the Akt1 gene in the heart resulted in adaptive cardiac hypertrophy with enhanced coronary angiogenesis and preserved contractility, whereas cardiac dilatation and failure was observed in response to chronic, long-term Akt1 stimulation (22). Similarly, VEGF-mediated proangiogenic therapy in mice was found to enhance neovascularization and to improve cardiac function, however, it could not prevent adverse remodeling and cardiac fibrosis characteristic of advanced disease stages (106). A higher expression of fetal cardiac genes and reduced cardiac performance after β-agonistic stress was also observed in mice with chronic increase in cardiac VEGF-A levels (64). These findings may explain why VEGF gene therapy is not established in clinical practice.

### Placental Growth Factor

VEGFR1-mediated cardioprotective effects have also been observed for placental growth factor (PlGF, also called PGF), a member of the VEGF superfamily secreted by cardiomyocytes and cardiac microvascular endothelial cells. Similar to VEGF, PlGF expression in the heart is regulated by HIF1α (107) and induced in myocytes and non-myocytes following pressure overload (108), ischemia/reperfusion injury in mice or oxygen glucose deprivation in murine neonatal cardiomyocytes (109). Mice with cardiac-specific inducible PlGF overexpression exhibited a more pronounced cardiac hypertrophy, greater increase in capillary density, but also an increased fibroblast content in the heart in response to pressure overload, whereas PlGF knockout mice died of heart failure within 1 week of pressure overload and showed an inability to upregulate angiogenesis (108). Of note, the cardiac phenotype of PlGF knockout mice was associated with increased cardiac tumor necrosis factor-α-converting enzyme expression and inflammation and could be completely rescued by *in vivo* RNA interference against tissue inhibitor of matrix-metalloproteinases-3 (110). PlGF overexpression in cardiac tissues was also found to be associated with increased endothelial-derived NO release which promoted cardiomyocyte hypertrophy by activating the Akt/mTORC1 pathway, whereas conditional cardiac-specific PlGF expression in eNOS knockout mice failed to induce myocardial hypertrophy (111). In addition, miR-182 was found to be upregulated in PlGF-treated mouse hearts, and treatment with anti-miR-182 prevented Akt/mTORC1 activation and inhibited the hypertrophic response (112). Of note, neither PlGF transgenic nor PlGF knockout mice exhibit any changes in cardiac angiogenesis at baseline (108, 113).

### Platelet-Derived Growth Factors

The expression of platelet-derived growth factors (PDGFs) is also markedly induced in the heart in response to load-induced stress (114), and studies in cultivated cells have shown that the expression of PDGFs and their receptors increases in response to hypoxia (115) or inflammation (116), both of which are present at increased levels in the hypertrophied heart.

The PDGF family consists of four ligands (PDGF-A to PDGF-D), which bind to PDGF receptor (PDGFR)-α or -β isoforms. While PDGFRα binds to PDGF-A, -B, and -C chains, the β-isoform of PDGFR binds only to PDGF-B and PDGF-D (117). Endothelial cells primarily express PDGF-B and PDGF-D (118) which signal via PDGFRβ receptors expressed on perivascular mesenchymal cells (smooth muscle cells, pericytes) (119) as well as cardiomyocytes (114).

PDGFs and their receptors have critical roles during development, including the heart and the vasculature (119). For example, mice lacking PDGFRα exhibit global connective tissue defects affecting the skin and many organs (120). Another striking feature in mice with defective PDGFRα signaling is the absence of pericytes resulting in aneurysmal dilation and hemorrhage (121). In this regard, factors governing pericyte contractility may participate in the regulation of angiogenic sprouting, as shown *in vitro* after gain- and loss-of-function modulation of the myosin phosphatase-RhoA-interacting protein in pericytes (122). Genetic deletion of PDGF-B or PDGFRβ in mice resulted in a complex cardiac phenotype with defects in ventricular septum formation and atrioventricular valve development as well as impaired coronary arteriogenesis (123, 124). Mice with genetic deletion of PDGFRβ in cardiomyocytes were found to be more susceptible to pressure overload and developed heart failure as a consequence of defects in stress-induced cardiac angiogenesis with subsequent fibrosis (114). The secretion of PDGF-B from endothelial cells and interaction with PDGFβ receptors expressed on myofibroblasts has also been shown to be critically involved in pericyte recruitment and vessel stabilization (125). Mice lacking PDGF-D exhibited a mild vascular phenotype with a disorganized cardiac vasculature and morphological changes in cardiac pericytes as well as an elevated blood pressure (118).

In addition to its role as upstream regulator of angiogenic signaling, PDGF may also contribute to the development of cardiac fibrosis. A systemic increase in PDGFRα kinase activity in mice leads to accelerated multi-organ fibrosis, including in the heart (126). Similarly, transgenic mice overexpressing PDGF-A in cardiomyocytes exhibited cardiac hypertrophy and fibrosis resulting in lethal heart failure shortly after birth (127). In contrast, the cardiac phenotype of PDGF-B overexpressing mice was less severe and consisted of focal areas of fibrosis and moderate cardiac hypertrophy (127). Genetically increased PDGFRβ activation was associated with increased proliferation and dedifferentiation of aortic vascular smooth muscle cells (128). Moreover, increased PDGFRβ signaling induced immune response signature genes in pericytes and mesenchymal cells. Overexpression of PDGF-C or -D under control of the α-myosin heavy chain promoter also induces cardiac fibrosis, hypertrophy and dilated cardiomyopathy in transgenic mice (129).

Importantly, administration of the small molecule tyrosine kinase inhibitor imatinib mesylate to mice was found to attenuate cardiac PDGFR phosphorylation and fibrosis induced by isoproteronol infusion (130). Similar findings were observed in a mouse model of TAC using the non-receptor tyrosine kinase inhibitor dasatinib (131). On the other hand, anti-cancer treatments with sunitinib malate targeting, among others PDGFRs, have been reported to cause left ventricular dysfunction and heart failure in a considerable number of patients (132, 133).

### Basic Fibroblast Growth Factor

Basic fibroblast growth factor (bFGF, also called FGF2) is also expressed in endothelial cells, including those in the heart, and has been shown to enhance myocardial collateral development in a canine model of coronary occlusion (134, 135). Later studies evaluated its proangiogenic role in the ischemic myocardium of rabbits (136), pigs (137), dogs (138), and mice (139) as well as in patients with severe ischemic heart disease (140). On the other hand, the role of FGF2 for cardiac vascularization during pressure overload induced hypoxia is less well-studied. For example, thyroxine, a potent stimulus of cardiac hypertrophy and vascularization, was shown to upregulate the expression of FGF2 and to increase cardiac capillary endothelial cell proliferation and angiogenesis (141). Mice with systemic FGF2 deficiency developed less severe cardiac hypertrophy in response to aortic banding (142) in line with the role of FGF2 as cardiomyocyte growth factor, whereas cardiac angiogenesis was not evaluated in this study. Studies also revealed differential effects of high and low molecular weight isoforms of FGF2 on cardiac hypertrophy, fibrosis and inflammation (143). Intramyocardial injection of FGF2 (plus heparin) in rats with hypertension-induced cardiac hypertrophy was associated with significant improvements in systolic pump function and ventricular dilation, as well as increased myocardial capillary density (144). Other members of the FGF growth factor family (e.g., acidic FGF or FGF1) may also promote angiogenesis (145) and have been shown to be overexpressed in hearts of patients with dilated cardiomyopathy (146), but their role in cardiac hypertrophy has not been directly examined experimentally. FGFs bind not only to FGF tyrosine kinase receptors, but with lower affinity also to heparan sulfate proteoglycans. In this regard, mice deficient in syndecan-4, a transmembrane proteoglycan and low-affinity receptor for FGF2, exhibited reduced capillary density, attenuated cardiomyocyte size and worsened left ventricular cardiac function after TAC surgery (147).

### Epidermal Growth Factors

Growth factors with potential angiogenic activities in the heart also include epidermal growth factor (EGF). Although earlier studies using rat heart endothelial cells revealed that EGF stimulated endothelial cells growth and colony formation (148), its contribution to cardiac angiogenesis under physiological and pathological conditions has not been extensively studied. Neuregulin-1 (NRG1) is also a member of the EGF family of growth factors expressed in the heart and has been shown to be involved in the regulation of cardiac development and repair by binding to ErbB (erythroblastic leukemia viral oncogene homolog) tyrosine kinase receptors on cardiomyocytes. Cardiotoxic side effects after treatment of cancer patients with monoclonal antibodies against erbB2 (trastuzumab) also suggested that the NRG/erbB pathway exerts cardioprotective effects under injury or stress (149). ErbB receptors are also expressed on endothelial cells, and recent data implicate NRG1 in the regulation of endothelial cell homeostasis, including blood pressure regulation and new vessel formation. Mice with inducible endothelial-specific deletion of NRG1 exhibited impaired neoangiogenesis and blood flow recovery following induction of unilateral hindlimb ischemia, and this phenotype could be reversed by recombinant human NRG1 (150). Regarding the heart, NRG1 was shown to promote cardiac angiogenesis, myocardial blood flood, and to improve left ventricular function in a rat model of streptozotocin-induced diabetic cardiomyopathy, directly via autocrine stimulation of endothelial erbB receptors and indirectly via increased expression of VEGF and angiopoietin-1 (151). Suppression of cardiac NRG1 levels, e.g., by sustained activation of β-catenin in endothelial cells, was shown to result in cardiac dysfunction, which could be rescued by recombinant NRG1 proteins (152). Via interaction with ErbB4 receptors on macrophages NRG1 may also suppress the release of proinflammatory cytokines and protect against cardiac fibrosis (153). These findings suggest that the NRG1/erbB pathway may be therapeutically useful to target cardiac angiogenesis, contractility and ultimately to treat patients with heart failure (154).

### Hepatocyte Growth Factor

Hepatocyte growth factor (HGF, also called scatter factor) and the tyrosine kinase receptor (c-Met) have also been implicated in myocardial function, both under physiological and pathological conditions (155). It has been shown *in vitro* that HGF directly promotes endothelial cell proliferation and migration (156) and inhibits apoptosis (157) resulting in new vessel formation in the ischemic heart (158) and skeletal muscle (159). In addition, HGF may indirectly exert proangiogenic effects via upregulation of VEGF expression in cardiomyocytes (160) and promoting its signaling activities (161). HGF also was found to downregulate the expression of VE-cadherin allowing endothelial migration, a necessary prerequisite for new blood vessel formation (162).

Factors modulating cardiac angiogenesis are not only produced by cardiomyocytes, but may also be derived from immune cells, in particular monocytes, as shown in mice after acute myocardial infarction (163). Furthermore, activated platelets may contribute to angiogenesis as a local source of VEGF, FGF, or PDGF and other growth factors, but they also store and release potent angiogenesis inhibitors, such as TGFβ, endostatin, angiostatin, or thrombospondin (164, 165). Although enhanced platelet activation has been reported in patients with cardiac hypertrophy and heart failure (166), the role of platelets for cardiac angiogenesis has not been directly examined. Also in mice, aortic banding was associated with higher platelet counts and increased platelet-leucocyte aggregates, and luminal platelet deposition was observed along the damaged endothelial cell layer (167).

### Endothelial Transcription Factors Regulating Angiogenic Growth Factor Expression

In addition to the transcriptional regulators ETS1, GATA2, and GATA4, mentioned above, other important factors controlling angiogenic gene transcription in endothelial cells include Kruppel-like factors (KLFs). The main KLFs expressed in endothelial cells are KLF2, 4, 6, and 15, which regulate the expression of a number of genes involved in endothelial cell homeostasis in response to changes in laminar blood flow, shear stress, or proinflammatory cytokines [reviewed in (168)]. KLF2 exerts anti-angiogenic effects by downregulating the receptor for VEGF (169) or inhibiting the hypoxia-induced expression of HIF1α and its proangiogenic target genes in endothelial cells (170). KLF4 regulates sprouting angiogenesis by complex interactions with ligands and receptors of the Notch signaling pathway (171), KLF6 by transcriptional induction of urokinase plasminogen activator in endothelial cells and subsequent activation of latent TGFβ (172). Whereas, deletion of KLF4 in cardiomyocytes was associated with increased mortality, myocardial fibrosis and cell death in response to TAC (173), its role in cardiac endothelial cells during hypertrophic remodeling has not been specifically addressed so far. Although KLF5 is not known to be expressed in endothelial cells, it has been shown to stimulate extracardiac angiogenesis and cardiovascular remodeling processes through activation of transcriptional regulators and nuclear receptors in smooth muscle cells (174).

**Figure 1:**
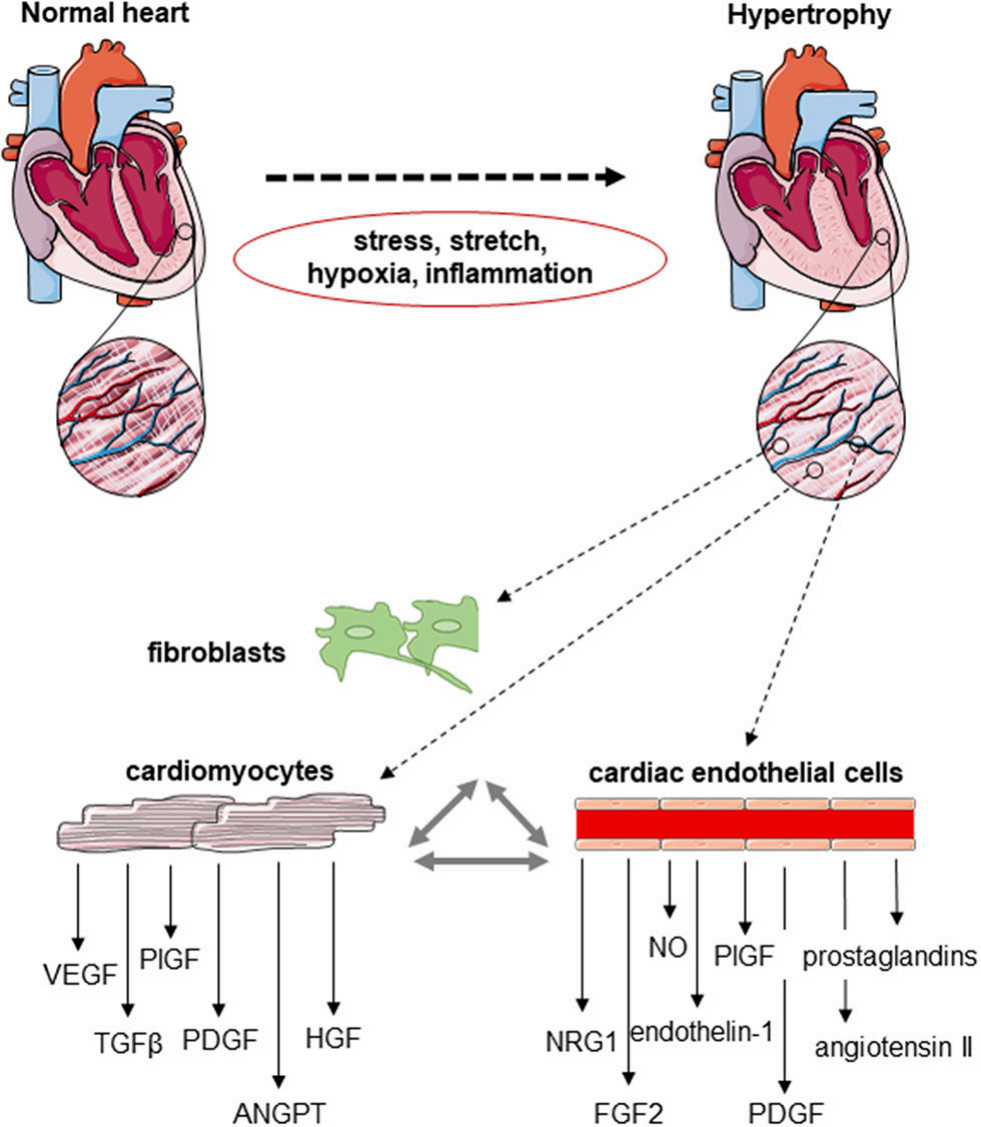
Schematic drawing of the cardiomyocyte–endothelial cell cross-talk during cardiac angiogenesis. Cardiomyocytes, fibroblasts and cardiac endothelial cells express a number of factors known to be involved in the regulation of endothelial cell proliferation, migration and new vessel formation, but also cardiomyocyte growth and contractility. The expression of these mediators of the endothelial–cardiomyocyte cross-talk changes in response to hemodynamic stress, mechanical stretch, hypoxia, and/or inflammation developing during cardiac hypertrophy.

Collectively, these data suggest a critical role of paracrine growth factors from cardiomyocytes and non-myocytes in modulating cardiac vascularization and the importance of a balanced cardiomyocyte and endothelial cell growth during cardiac hypertrophy. A schematic drawing depicting some of the changes during the development of cardiac hypertrophy is provided in Figure 1. However, the important question remains: why do angiogenesis defects develop despite increased hypoxia and energy demands?

## Possible Mechanisms Underlying the Reduced Cardiac Angiogenesis During Pathological Hypertrophy

Whereas, the role of VEGF and other angiogenic growth factors for cardiac angiogenesis has been intensively studied, the molecular mechanisms and mediators underlying the rarefaction of the cardiac microvasculature during pathological hypertrophy are less well-understood. The existing clinical and preclinical evidence suggests that they may include an insufficient vessel growth due to inadequate release of angiogenic growth factors from cardiomyocytes, platelets and inflammatory cells, impaired angiogenic signaling and the regression, or death of cardiac endothelial cells.

### Imbalance of Pro- and Anti-angiogenic Growth Factors in the Heart During Hypertrophy

**Figure 2:**
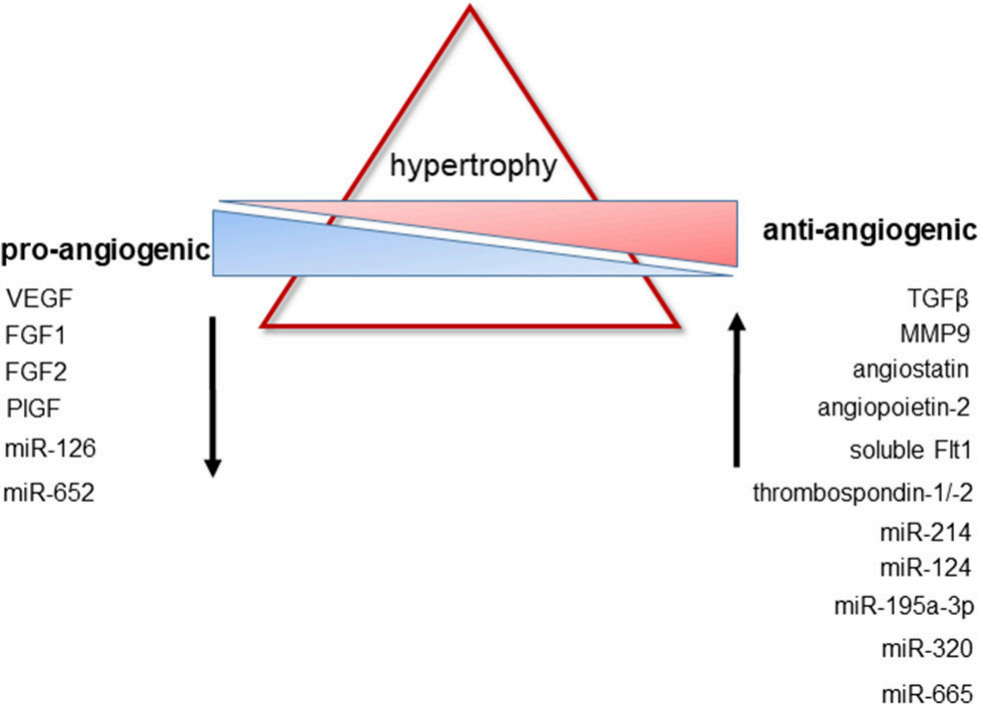
Changes in pro- and anti-antiangiogenic factors during cardiac hypertrophy. Pathological hypertrophy is associated with a decreased expression of factors known to promote new vessel formation, whereas the expression of factors with anti-angiogenic properties is upregulated. For further details and additional regulatory mechanisms, please see the text.

A reduced cardiac expression of VEGF has been reported in patients with dilated cardiomyopathy and heart failure (175), whereas levels of the VEGF antagonist soluble VEGFR1 were found to be increased in pressure-overloaded myocardium of rabbits (176). Changes in myocardial elasticity and an increased myocardial stiffness due to hypertrophic remodeling may alter the synthesis and release of VEGF, as suggested by atomic force microscopical findings in cardiomyocytes (177). At least *ex vivo*, pretreatment with pulsed magnetic field stimulation to increase endothelial VEGF and FGF2 secretion was successful to accelerate proliferation and migration of cardiac microvascular endothelial cells isolated from rat hearts (178), and may be further explored for its efficacy in restoring the properties of endothelial cells from hypertrophied or dilated hearts. TGFβ, an inhibitor of endothelial cell proliferation (179), is expressed at elevated levels in myocardial hypertrophy particularly during the transition from compensated cardiac hypertrophy to heart failure (180–183). Downstream mediators of TGFβ signaling have been shown to induce the production of anti-angiogenic factors, such as thrombospondin-1, in case of Smad2 and Smad4 (184, 185), or to promote angiogenesis via stimulation of VEGF expression, in case of Smad3 (186). Of note, Smad transcription factors not only regulate the cellular effects of TGFβ, but also of other ligands involved in angiogenesis, including bone-morphogenic proteins (180). Moreover, TGFβ receptors have been shown to synergize with other receptors involved in the regulation of angiogenesis, including Notch (182) and COUP-TFII (183). The increased expression of matrix-metalloproteinase-9 observed in hypertrophic hearts of rabbits after aortic banding may also contribute to the inadequate capillary growth during cardiac hypertrophy via the proteolytic generation of endogenous angiogenesis inhibitors, such as angiostatin, endostatin, or tumstatin (187). Elevated levels of endostatin and the angiogenesis inhibitors angiopoietin-2 and thrombospondin-2 were also detected in serum of patients with heart failure due to chronic kidney disease (188). Elevated levels of the VEGF inhibitor soluble Flt1 (VEGFR1) were observed in plasma samples of women with peripartum cardiomyopathy, and exogenous administration of soluble Flt1 into mice produced diastolic dysfunction (189). Please also see Figure 2 for changes in pro- and anti-angiogenic factors during cardiac hypertrophy. On the other hand, elevated VEGF levels have been detected in serum of patients with dilated cardiomyopathy (190) suggesting that an impaired responsiveness of coronary endothelial cells toward angiogenic growth factor stimulation may also be involved in the observed rarefaction of the cardiac microvasculature. Similar findings were observed for serum PlGF levels in patients with ischemic heart failure (191). In a mouse model of dilated cardiomyopathy, cardiac VEGF-A expression was found to be upregulated without a parallel increase in cardiac angiogenesis (192). Of note, biomarkers of angiogenesis, such as neuropilin, were shown to predict the outcome in patients with heart failure and preserved ejection fraction, but not in those in whom cardiac output was already reduced (193).

### Endothelial Phosphatases as Mediators of Angiogenic Resistance

In addition to an inadequate expression of angiogenic growth factors by hypertropied cardiomyocytes, upregulation of counterregulatory mediators and resistance toward angiogenic growth factor stimulation may also contribute to the inadequate angiogenesis observed during pathological cardiac hypertrophy. In this regard, several growth factors involved in the modulation of cardiac angiogenesis are known to signal via tyrosine kinase receptors, whose activity is negatively regulated by protein tyrosine phosphatases (PTP) and the dephosphorylation of specific phosphotyrosine residues. In addition, PTPs may also control the phosphorylation state of intermediary signaling proteins involved in growth factor and integrin signaling, endothelial cell function and angiogenesis.

A major PTP expressed in endothelial cells is Protein Tyrosine Phosphatase (PTP)-1B. PTP1B is overexpressed in human and murine heart failure: For example, a significantly increased PTP1B activity was detected in hypertrophic hearts of rats after bandings as well as in left ventricular tissue specimens of patients with systolic dysfunction undergoing aortic valve replacement (194), and we have shown in mice that cardiac hypertrophy is associated with elevated cardiac PTP1B protein levels (195). Potential mechanisms underlying increased PTP1B expression during cardiac hypertrophy include ischemia (196) and inflammation (197), and our finding that PTP1B overexpression coincided with elevated HIF1α levels suggested that cardiac hypoxia may also play a role (195). Interestingly, and although PTP1B is ubiquitously expressed, deletion of PTP1B selectively in endothelial cells markedly reduced cardiac PTP1B levels underlining the importance of endothelial cells as a major source of PTP1B in the heart, at least following TAC (195).

In line with the role of PTP1B as negative regulator of VEGFR2 autophosphorylation (196), we could show that inducible deletion of PTP1B in endothelial cells improves cardiac angiogenesis and reduces cardiac hypoxia, but also ameliorates oxidative stress and fibrosis resulting in an improved survival and better cardiac pump function (195). Endothelial cell-specific deletion of PTP1B enhances VEGF signal transduction and new vessel formation also under normal conditions or in response to skeletal muscle ischemia, as shown in mice (198). Previous studies examined the role of PTP1B in mouse models of chronic myocardial ischemia and could show that systemic pharmacological inhibition or global gene deletion of PTP1B protects mice against chronic heart failure induced by myocardial infarction (199, 200).

Elevated levels of PTP1B have also been implicated in the pathophysiology of endothelial dysfunction. Not only may endothelial failure to vascularize the heart contribute to cardiac decompensation, but heart failure itself is associated with endothelial dysfunction. In this regard, pharmacological inhibition of PTP1B was shown to improve the peripheral endothelial dysfunction in mice with post-ischemic heart failure (201). Enhanced NO production and improved endothelial dysfunction was also observed in mice with constitutive PTP1B gene deletion in Tie2-expressing cells and heart failure following myocardial infarction (202). Moreover, genetic inactivation of PTP1B has been shown to increase endothelial NO release and to ameliorate cardiac dysfunction in mice with age-associated heart failure (203). All of these findings suggest that inhibition of PTP1B could be a promising approach to prevent or treat endothelial dysfunction and defects in cardiac angiogenesis in patients with heart failure. In this regard, microRNA-210 targeting PTP1B was shown to improve angiogenesis, to inhibit apoptosis and to improve cardiac function in a mouse model of myocardial infarction (204).

In addition to VEGF receptors, other receptor tyrosine kinases expressed in the heart and regulated by PTP1B include receptors for PDGF, FGF, EGF, or HGF. PTP1B may also dephosphorylate Tie1 and Tie2, i.e., the receptors for angiopoietin-1 and -2, major angiogenic growth factors involved in vessel maturation and stabilization (60), although this has not been examined so far. Dephosphorylation of the insulin receptor by PTP1B may result in cardiac insulin resistance and lead to mitochondrial and contractile dysfunction, as shown in a rat model of pressure overload-induced heart failure (194). However, others have shown that excessive cardiac insulin signaling exacerbates systolic dysfunction induced by pressure overload in rodents (205). Dephosphorylation of the leptin receptor by PTP1B may also affect cardiac function, however the consequences may depend on the cell type and the presence and duration of local (e.g., during cardiac hypertrophy) or systemic leptin overexpression (e.g., in obesity) (206–209).

A genome-wide gene expression analysis of mouse hearts revealed that increased cardiac afterload is associated with elevated expression of several counterregulatory phosphatases (51), including PTP1B as well as others. Phosphatases potentially relevant for cardiac endothelial cell signaling include receptor-type tyrosine-protein phosphatase-beta (210), phosphatase and tensin homolog (211) or the serine-threonine phosphatases PP1 (212–214) and PP2A (215) or lipid phosphate phosphatase-3 (216). Loss-of-function mutations in PTPN11 [encoding protein tyrosine phosphatase Src homology domain-2 containing tyrosine phosphatase [SHP2]] have been implicated in hypertrophic cardiomyopathy associated with Noonan syndrome, and endothelial-specific expression of SHP2 was sufficient to induce adult-onset cardiac hypertrophy in mice (217). Interestingly, a combined gene delivery strategy using hypoxia-inducible plasmids expressing VEGF, hemeoxygenase-1 and a miRNA targeting SHP1 were found to synergistically enhance cardiac angiogenesis and to prevent apoptosis (218). Changes in cardiac phosphatase activities may also indirectly affect cardiac angiogenesis, as shown in mice lacking PH domain leucine-rich repeat protein phosphatase: The resulting activation of protein kinase B in cardiomyocytes was found to increase VEGF expression and to improve cardiac angiogenesis, both at baseline and after TAC-induced pressure overload (219).

**Figure 3:**
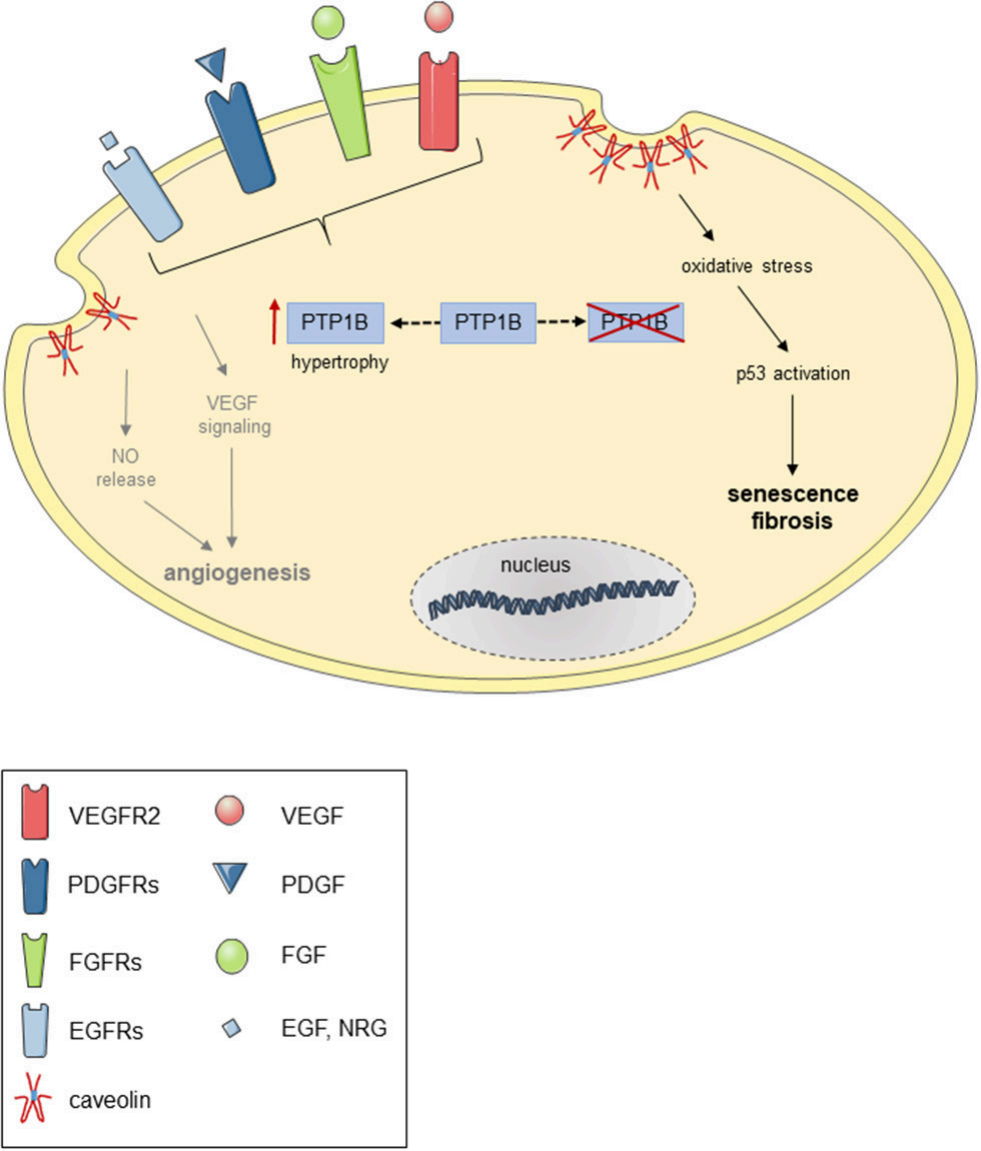
Possible consequences of PTP1B overexpression or deletion in endothelial cells. Hypothetical scheme demonstrating possible consequence of PTP1B overexpression (e.g., during cardiac hypertrophy) and pharmacological or genetic inactivation of PTP1B in endothelial cells on receptor tyrosine kinase-mediated NO release and angiogenic signaling and oxidative stress-induced p53 activation and cellular senescence.

On the other hand, unrestricted growth factor signaling in the absence of counterregulatory mechanisms may also not be desirable, as recent findings in obese mice from our group suggest (220). In this study, we observed that absence of PTP1B in endothelial cells was associated with signs of premature endothelial cell senescence, resulting in impaired reendothelialization of carotid artery lesions induced by chemical vascular injury. Mechanistically, we could show that genetic deletion or siRNA-mediated knockdown of PTP1B was associated with increased expression of the PTP1B substrate caveolin-1 and enhanced oxidative stress, and siRNA-mediated downregulation of caveolin-1 or “treatment” with an antioxidant prevented endothelial senescence and improved *in vitro* reendothelialization. Replicative senescence and premature telomere shortening due to continuous receptor tyrosine kinase overstimulation may also have played a role, as shorter telomeres have been observed in leucocytes (221) and cardiomyocytes (222) of patients with heart failure, independent of age. In mice, absence of telomerase was associated with attenuated cardiac myocyte proliferation, increased apoptosis and cardiac myocyte hypertrophy and resulted in cardiac dilatation and heart failure (223). A hypothetical schema of the potential consequence of PTP1B overexpression or deletion in endothelial with respect to receptor tyrosine kinase signaling, angiogenesis and senescence is shown in Figure 3.

### Cardiac Endothelial Cell Death as Possible Cause Underlying Vascular Rarefaction

Increased endothelial cell death may also contribute to the rarefaction of the cardiac vasculature observed during pathological hypertrophy. In fact, angiogenic growth factors may act by inhibiting apoptosis (224), and promotion of endothelial cell survival represents a central mechanism during new vessel formation (225). Increased p53 expression and other components of apoptosis pathways have been observed in myocardial biopsies of patients with heart disease and were found to progressively increase during transition toward heart failure (226–228), and similar findings were obtained in rats following TAC (229). Quantitative analysis revealed that a considerable number of endothelial cells undergo apoptotic cell death after TAC (230), and pressure overload in mice was found to be associated with increased endothelial expression of tumor suppressor p53, a central regulator of apoptosis and cell cycle arrest (231). In a rat and a canine model of left ventricular hypertrophy and heart failure following chronic aortic banding, the overwhelming majority of apoptotic cells was identified as non-cardiomyocytes (232). In line with these observations, systemic caspase inhibition had no effect on the cardiomyocyte number, but enhanced angiogenesis to reduce cardiac fibrosis and to augment cardiac contractility following TAC injury in mice (233). We have demonstrated the importance of p53 expressed in endothelial cells for maintaining cardiac vessel density during hypertrophy, but also for angiogenesis in response to ischemia induced by unilateral femoral artery ligation (234). Previous studies had shown that global p53 deficiency (45, 235, 236) or pharmacological p53 inhibition (237) protect against cardiac injury, whereas activation of p53 following deletion of the p53 inhibitor mdm2 in cardiomyocytes accelerated left ventricular function deterioration in response to aortic banding or myocardial infarction (238). Similar cardioprotective effects were observed in mice with global deletion of Puma (p53-upregulated modulator of apoptosis), a proapoptotic Bcl-2 family protein that serves as general sensor of pathological apoptotic stimuli (239). Based on the above findings identifying p53 accumulation in the heart as essential mechanism and characteristic of cardiac decomponsation, AAV vectors containing the VEGF gene driven by a p53-responsive promoter have been tested in rats and were found to improve cardiac function, to reduce fibrosis and to reverse capillary rarefaction (240). Mice expressing heat shock transcription factor-1 (HSF-1) exhibiting reduced endothelial p53 expression were characterized by increased angiogenesis and cardiac HIF1α expression and protected against pressure overload-induced heart failure, whereas HSF1 deficient mice exhibit aggravated cardiac remodeling under pressure overload due to impaired, imbalanced angiogenesis (241).

A p53-mediated impairment of cardiac angiogenesis was also observed in other models of cardiac hypertrophy and inflammation, such as following stimulation with angiotensin II (242). Moreover, senescence-accelerated mice, a model of aging, displayed endothelial dysfunction and developed cardiac hypertrophy and diastolic dysfunction associated with increased endothelial senescence, as shown by acetyl-p53 and CD31 co-staining (243). In this regard, members of the mammalian sirtuin (Sirt) family of NAD-dependent histone deacetylases (HDACs) involved in the deacetylation and inactivation of p53, have been shown to ameliorate cardiac apoptosis and cell death and to protect the heart (244). The potential relevance of mechanisms of epigenetic regulation is underlined by findings in aged mice with Sirt1 deletion in endothelial cells exhibiting diastolic dysfunction and decreased cardiac angiogenesis as well as an impaired *ex vivo* response to VEGF (245). Conversely, endothelial cell-specific Sirt3 transgenic mice showed decreased fibrosis as well as improved cardiac function and microvascular network compared with wild-type mice stimulated with angiotensin II for 2 weeks (246). Since the accumulation of p53 and the decline of sirtuins is a hallmark of aging, an established risk factor of heart failure, these mechanisms may be particularly important for the age-associated rarefaction of the cardiac vasculature, mentioned above.

Tumor suppressor p53 may accumulate in the heart during sustained pressure overload as a consequence of hypoxia and elevated HIF1α levels (45). P53 promotes the ubiquitination and proteosomal degradation of HIF1α resulting in reduced expression of angiogenic growth factors despite the presence of hypoxia and a critical mismatch between myocardial growth and capillary growth (45, 247). The inability to stabilize HIF1α and to maintain the expression VEGF and other angiogenic growth factors may contribute to vascular rarefaction during prolonged phases of hypoxia and promote the transition toward heart failure. In this regard, the small Rho GTPase Rnd3 was shown to physically interact with HIF1α and to prevent its degradation (248). Moreover, mice with genetic deletion of Rnd3 developed dilated cardiomyopathy after aortic banding, whereas cardioprotective effects were seen in Rnd3 transgenic mice. In patients with end-stage heart failure, Rnd3 expression was found to be depressed. Also, dietary copper supplementation was shown in mice to activate HIF1α and enhance VEGF expression in a mouse model of hypertrophic cardiomyopathy (249).

Regarding the mechanisms how p53 may inhibit angiogenesis, in addition to its role in apoptosis, a previous study had shown that p53 transcriptionally activates the a(II)-collagen prolyl-4-hydroxylase gene resulting in the extracellular release of antiangiogenic fragments from collagen type 4 and 18 (250). Others found that p53 activates the promoter of the thrombospondin-1 (TSP1) gene (251), an endogenous inhibitor of angiogenesis expressed in endothelial cells (252). In line with p53 accumulation during cardiac hypertrophy, pressure overload is associated with increased cardiac expression of TSP1 (253) and TSP4 (254), and the differential expression of proangiogenic (e.g., VEGF) and antiangiogenic (e.g., TSP1) growth factors in the hypertrophied heart during chronic hypoxia may contribute to the suppressed angiogenesis observed during late stages of cardiac hypertrophy, as suggested by findings in a rat model of angiotensin II-induced cardiac hypertrophy and heart failure (255). In addition, TSP1 may also activate latent TGFβ (256), a pleiotropic growth factor with anti-angiogenic and profibrotic properties. In addition, p53 may also regulate other processes relevant for the heart, including inflammation (231), and deletion of p53 from endothelial cells reduced signs of endothelial cell activation and decreased cardiac inflammation (227). Apoptotic endothelial cell damage and release of cell membrane microparticles may also contribute to cardiac inflammation (257).

### Epigenetic Control of Angiogenic Gene Transcription

The expression of angiogenic growth factors during cardiac hypertrophy may also be regulated on the level of chromatin structure and the acetylation of histone proteins (but also other proteins including transcription factors) by either histone acetyl transferases (HATs) or histone deacetylases (HDACs) resulting in activation or inhibition of gene transcription, respectively. These and other mechanisms involved in the epigenetic control of gene expression in cardiac hypertrophy and heart failure have also been very clearly presented in a recent review article to which we refer for further details (258). In fact, HDAC inhibitors have already been successfully tested in preclinical models of heart failure [recently reviewed in (259)]. Histone deacetylases involved in the regulation of endothelial cell function include HDAC1, which has been shown to decrease basal and endothelin-1 stimulated NO production via eNOS protein deacetylation (260), whereas HDAC2 upregulates the release of NO by decreasing arginase-2 gene expression (261, 262). HDAC3 may regulate VEGF-induced endothelial differentiation by deacetylation of p53 and p21 activation (263). A role for HDACs in the positive regulation of angiogenesis has been shown for HDAC6 (264), HDAC7 (265), and HDAC9 (266), whereas HDAC5 inhibits endothelial sprouting by repression of angiogenic guidance factors (267). Of note, general inhibitors of HDACs, such as trichostatin A, butyric or valproic acid, may decrease endothelial differentiation and the lineage commitment of progenitor cells (268, 269), and epigenetic control mechanisms may play a role in the endothelial cell transition toward the mesenchymal lineage (270), a process involved in cardiac vessel rarefaction and fibrosis (271). Moreover, many HDACs are ubiquitously expressed, including cardiomyocytes and non-myocytes, and HDAC5 or HDAC9 deficiency in mice was associated with cardiac hypertrophy (272). Spontaneous cardiac hypertrophy with simultaneous compensatory blood vessel growth due to angiogenic gene induction was observed in mice with myocyte-restricted expression of the histone acetyltransferase p300 (273, 274).

Other mechanism involved in the regulation of angiogenic gene transcription and vascular development include chromatin remodeling enzymes, such as the ATPases Brahma-related gene-1 and Brahma (275) or Ino80 (276), which act by modulating the accessibility of chromatins. Ino80 has been shown to be essential for coronary angiogenesis in developing mouse hearts, but whether this or other chromatin remodelers play a role during pathological remodeling processes in the adult heart remains to be shown.

## Endothelial Cell Cross-Talk With Other Cardiac Cell Types

Endothelial cells are not only important cellular mediators of cardiac vascularization, but are also actively involved in communicating with adjacent cardiomyocytes, fibroblasts and other cells resident in the heart by the release of growth factors, such as NO (277–280), endothelin-1 (40, 277), angiotensin II, prostaglandins or neuregulin-1 (277, 281, 282) (Figure 1). In fact, the interplay of endothelial cells with their local environment modulates cardiac remodeling processes, and it is the balance of positive and negative signals that determines the outcome of the remodeling response. Of note, factors promoting cardiomyocyte hypertrophy do not necessarily also promote endothelial cell growth and proliferation. For example, endothelin-1 promotes cardiac hypertrophy, but also is a well-known anti-angiogenic agent (283). Since the factors secreted by endothelial cells affecting cardiac myocyte and non-myocyte morphology and function including contractility and fibrosis have been recently presented in two excellent review articles (40, 282), we will not further discuss these aspects, but refer the reader to those and other previous publications.

## Role of Endothelial Cells in Cardiac Fibrosis

Cardiac fibrosis is an important histological feature of pathological hypertrophy, and the accumulation of extracellular matrix proteins contributes to ventricular dilatation and contractile dysfunction. Mediators expressed at increased levels in hypertrophied hearts involved in fibroblast activation and cardiac fibrosis include aldosterone, angiotensin II, endothelin-1 as well as the growth factors TGFβ1, PDGFs, and connective tissue growth factor, among others (284). In addition to (myo)fibroblasts, endothelial cells also may undergo phenotypic changes in response to pressure overload and transdifferentiate into myofibroblast-like cells with increased production of extracellular matrix proteins. This process is termed endothelial-to-mesenchymal transition (EndMT) and has been shown to play a role in the development of kidney (285), lung (286), and cardiac fibrosis (271), among others. In rat hearts with aortic constriction-induced cardiac hypertrophy, local differences in cardiomyocyte hypertrophy and collagen deposition were observed which correlated with local reduction in capillary density (287). Hypoxia was shown to induce phenotypic changes consistent with EndMT, and HIF1α-induced induction of the transcription factor SNAIL in coronary artery endothelial cells has been identified as potential mechanism linking hypoxia and fibrosis in the heart (288). In line with the role of p53 in HIF1α degradation (45), we could show that endothelial deletion of p53 attenuated cardiac fibrosis and was associated with lower mRNA expression of transcription factors controlling mesenchymal differentiation, both in banded mouse hearts and primary endothelial cells stimulated with TGFβ1 (234), a profibrotic growth factor and prototypical inducer of EndMT (289). Hypoxia may induce the expression of TGFβ1, and increased expression levels of TGFβ1 have been observed during pathological cardiac hypertrophy remodeling (21) including TAC (290) and angiotensin II (291) or isoproteronol (292) stimulation. Overexpression of TGFβ was also observed in patients with idiopathic hypertrophic cardiomyopathy (293). Importantly, TGFβ1 expression was found to increase particularly during the transition from stable to symptomatic heart failure (294). TGFβ1 transgenic mice develop cardiac hypertrophy and interstitial fibrosis already under baseline conditions (295). Regarding cardiac angiogenesis, TGFβ was shown to inhibit FGF-induced endothelial cell proliferation (179, 296) and tube-like structure formation (297). *In vivo*, TGFβ1 released from activated platelets inhibited endothelial regeneration (298) and promoted apoptosis in TNF-activated human brain endothelial cells (299), whereas mice lacking TGFβ1 in platelets were characterized by improved restoration of the endothelial layer covering a neointima (300), suggesting that increased cardiac levels of TGFβ may contribute to cardiac vessel rarefaction.

Not only the amount, but also the composition of the extracellular matrix may play a role in the regulation of endothelial cell phenotypes, for example via interaction with integrins expressed on endothelial cells. Syndecan-4, a heparan sulfate-carrying core protein expressed in cardiomyocytes and present in the extracellular matrix of the heart, binds several growth factors, including FGF2. On the other hand, matrix-metalloproteinase or other proteases may release anti-angiogenic factors from extracellular matrix proteins and thus disturbed the balance of factors controlling vessel growth. Any changes in the composition developing during cardiac hypertrophy remodeling may alter angiogenic signaling and endothelial responsiveness and contribute to the functional and structural deterioration.

## Concluding Remarks

During the past years, considerable knowledge has accumulated regarding the regulation of new vessel formation in the healthy and the hypertrophied heart. It has become clear that cardiac endothelial cells not only respond to hemodynamic forces and paracrine signals from neighboring cells, but also actively participate in cardiac remodeling processes by stimulating cardiomyocyte growth and contractility or the production of extracellular matrix proteins in myofibroblasts. Moreover, in response to adequate signals they may change their phenotype and transdifferentiate into extracellular matrix-producing cells. Because cardiac vascularization plays a central role in the transition from adaptive cardiac hypertrophy to heart failure, endothelial cells and signaling mechanisms involved in the regulation or dysregulation of angiogenesis in the heart represent promising therapeutic targets to improve pressure overload-induced cardiac remodeling and to prevent the transition to heart failure. However, potential treatment strategies are currently limited to the experimental, preclinical stage and have not entered the clinic.

## Author Contributions

RG, MB, and KS wrote the manuscript. KS acquired funding. Authors have read and approved the final manuscript.

### Conflict of Interest Statement

The authors declare that the research was conducted in the absence of any commercial or financial relationships that could be construed as a potential conflict of interest.
